# Health-related quality of life of long-term adolescent and young adult (AYA) cancer survivors compared to a matched normative population: results of the SURVAYA study

**DOI:** 10.1007/s11764-025-01818-0

**Published:** 2025-06-17

**Authors:** Silvie H. M. Janssen, Carla Vlooswijk, Rhodé M. Bijlsma, Suzanne E. J. Kaal, Jan Martijn Kerst, Jacqueline M. Tromp, Monique E. M. M. Bos, Tom van der Hulle, Roy I. Lalisang, Janine Nuver, Mathilde C. M. Kouwenhoven, Winette T. A. van der Graaf, Olga Husson

**Affiliations:** 1 Department of Medical Oncology, Netherlands Cancer Institute – Antoni van Leeuwenhoek, 1066 CX Amsterdam, The Netherlands; 2 Research and Development, Netherlands Comprehensive Cancer Organization, 3511 DT Utrecht, The Netherlands; 3 Department of Medical Oncology, University Medical Center Utrecht, 3584 CX Utrecht, The Netherlands; 4 Department of Medical Oncology, Radboud University Medical Center, 6525 GA Nijmegen, The Netherlands; 5 Department of Medical Oncology, Amsterdam University Medical Centers, 1105 AZ Amsterdam, The Netherlands; 6 Department of Medical Oncology, Erasmus MC Cancer Institute, Erasmus University Medical Center, 3015 GD Rotterdam, The Netherlands; 7 Department of Medical Oncology, Leiden University Medical Center, 2333 ZA Leiden, The Netherlands; 8 Division of Medical Oncology, Department of Internal Medicine, UMC+ Comprehensive Cancer Center, GROW-School of Oncology and Reproduction, Maastricht University Medical Center+, 6229 HX Maastricht, The Netherlands; 9 Department of Medical Oncology, University Medical Center Groningen, 9713 GZ Groningen, The Netherlands; 10 Department of Neurology, Amsterdam UMC, Cancer Center Amsterdam, Amsterdam University Medical Centers, Location VUmc, 1081 HV Amsterdam, The Netherlands; 11 Department of Surgical Oncology, Erasmus MC Cancer Institute, Erasmus University Medical Center, 3015 GD Rotterdam, The Netherlands; 12 Department of Public Health, Erasmus MC Cancer Institute, Erasmus University Medical Centre, 3015 GD Rotterdam, The Netherlands

**Keywords:** Adolescents and young adults, AYAs, Cancer, Survivorship, Health-related quality of life, EORTC QLQ-C30

## Abstract

**Purpose:**

Health-related quality of life (HRQoL) is a commonly assessed patient-reported outcome that might be especially relevant for the adolescent and young adult (AYA) cancer survivor population. However, limited data is available regarding the HRQoL of long-term AYA cancer survivors compared to a matched normative population and associated factors.

**Methods:**

AYA cancer survivors (18–39 years at initial diagnosis; 5–20 years post-diagnosis), identified by the Netherlands Cancer Registry (NCR), were invited for participation in the SURVAYA questionnaire study. Participants self-reported their socio-demographics, health-related conditions, healthcare use, positive life outlook, and HRQoL (EORTC QLQ-C30). An age- and sex-matched normative population was randomly composed. Clinical data were retrieved from the NCR.

**Results:**

A total of 3745 AYAs (on average 32 years old at diagnosis, 12 years post-diagnosis) and 517 peers without cancer (normative population) were included. All functioning scales showed significant differences, with AYA cancer survivors scoring lower: cognitive (77.9 vs. 92.2), role (83.2 vs. 91.3), social (87.9 vs. 93.6), physical (91.5 vs. 94.3), and emotional functioning (79.5 vs. 83.4). No significant difference was observed in global QoL. Female sex, lower educational attainment, older age at diagnosis, several tumor types, radiotherapy, chemotherapy, higher stage, more health-related conditions, and more healthcare visits were negatively associated with several HRQoL scales.

**Conclusions:**

AYA cancer survivors face worse HRQoL compared to peers in all functioning domains, but it is most pronounced in cognitive functioning. This study underlines the need for timely cancer survivorship care to regain, improve, and ensure the quality of life of current and future AYA cancer survivors.

**Clinical trial registration.:**

NCT05379387.

**Supplementary Information:**

The online version contains supplementary material available at https://doi.org/10.1007/s11764-025-01818-0.

## Introduction

Patient-reported outcomes (PROs) have moved to the forefront of cancer research and clinical care over the years. They provide patients the opportunity to self-rate and express the impact of a cancer diagnosis and/or treatment in addition to clinical measures. A commonly used PRO is health-related quality of life (HRQoL). HRQoL is a multidomain concept covering the patient’s perception of the impact of illness (treatment) on life’s physical, psychological, and social aspects [1].

HRQoL assessment is especially relevant for the vulnerable adolescent and young adult (AYA) cancer population [2]. AYAs, who are 18–39 years old at their first cancer diagnosis, are stuck between pediatric and adult oncology [3, 4]. Besides their age, AYAs distinguish themselves from pediatrics and older adults in their clinical characteristics, such as tumor types, biology, and treatment, as well as their care needs and developmental life phase [3, 5]. The latter refers to the age-specific milestones which are characteristic for this age group, including career development, (financial) independence, family formation, and home ownership. The unique and challenging milestones characteristic of the AYA population could influence the impact of cancer (treatment) on HRQoL differently compared to pediatric and older adult cancer populations [2].

The number of AYA cancer survivors is rising worldwide, as the incidence of cancer in and the overall 5-year relative survival of AYAs has increased over time [3, 6]. As AYA survivors have an overall 5-year relative survival of > 85% and, therefore, a large part of their lives still ahead of them, it is important to ensure both the quantity as well as quality of their survival [6]. While most survivors yearn for a *new normal life* after cancer, this is often complicated by the aftermath and effects of cancer (treatment) [7–9]. These effects may develop in the short term (during or right after treatment), such as lymphedema and fatigue, but also in the long term (years after diagnosis), such as cardiovascular problems and depression [3, 10]. Altogether, these factors may significantly impact HRQoL.

Previous studies on HRQoL among AYA cancer survivors show poorer (physical and mental) HRQoL scores for AYAs compared to normative populations [11–14]. However, some studies show conflicting results when comparing the HRQoL of AYAs with a normative population or differences in HRQoL when stratified for sociodemographic or clinical characteristics, like sex, age, and treatment type [15–20]. For example, in the study of Harju et al., male AYA cancer survivors reported better mental health than controls, in contrast to female AYAs, while Murnane et al. showed no difference in overall mental health between AYAs and the normative population [15, 16]. Jörngården et al. showed that HRQoL levels reversed over time, leading to significantly better HRQoL for AYAs compared to the normative population 1.5 years after diagnosis [17]. However, many studies focusing on the HRQoL of AYA cancer survivors are limited by either small study populations [16, 17, 21], limited coverage of the AYA tumor epidemiology [14, 22], limited age span (i.e., mostly focusing on AYAs who are 25 years or younger at diagnosis) [15–18, 20, 21, 23, 24], short-term survivorship (i.e., mostly focusing on the first 5 years following diagnosis/end of treatment) [13, 14, 16, 17, 19–25], or a lack of a matched normative population [19, 22, 25]. The only studies that were not limited by the aforementioned characteristics have been performed in North America and used measurement tools yielding subscale scores that focus mainly on physical and mental health and general HRQoL [11, 12, 26].

The aims of this cross-sectional Dutch study were to (1) compare HRQoL of long-term AYA cancer survivors with HRQoL of an age- and sex-matched normative population and (2) determine which factors (sociodemographic, clinical, healthcare use, and positive life outlook) are associated with AYA cancer survivors’ HRQoL. Based on this data, we strive to create input for optimizing and tailoring evidence-based support and guidelines for current and future AYA cancer survivors. Within this study, long-term AYA cancer survivors are defined as adolescents and young adults who are 18–39 years old at initial cancer diagnosis and 5–20 years post cancer diagnosis.

## Methods

### Data collection

This study presents a primary data analysis of the SURVAYA study, a nationwide, retrospective, observational questionnaire study to examine, among others, the HRQoL of long-term survivors of cancer in adolescence and young adulthood (clinical trial registration NCT05379387). The methodology of the SURVAYA study has been described previously in detail [27]. Briefly, the Netherlands Cancer Registry (NCR) identified all AYAs at the age of 18–39 years who were diagnosed with their first malignancy (the Dutch AYA definition) between 1999 and 2015 in one of the nine Dutch participating treating centers. As the Dutch cancer care is divided into care for those < 18 years at cancer diagnosis (pediatric oncology) and ≥ 18 years at cancer diagnosis (medical oncology), the lower age limit of AYAs in the SURVAYA study was set to 18 years instead of 15 years. The records identified by the NCR were linked with the Dutch municipal records database to ascertain whether the AYAs were still alive at the moment of invitation and to obtain their current addresses. All eligible AYAs then received an invitation from their (former) medical specialist regarding their potential participation in the SURVAYA study. If willing to participate, AYAs could complete the questionnaire either online or on paper, after signing the informed consent form using the PROFILES registry (*Patient Reported Outcomes Following Initial treatment and Long-term Evaluation of Survivorship*) [28]. Data was collected between May 2019 and June 2021. Once completed, the questionnaire data were linked with the clinical data of the NCR. The Institutional Review Board of the Antoni van Leeuwenhoek – Netherlands Cancer Institute approved the study (IRBd18122).

### Normative population

CentERdata, a Dutch institute for data collection and research of Tilburg University, regularly obtains data regarding a range of outcomes of a reference panel, which represents a normative cohort of the Dutch-speaking population in the Netherlands. Members of this normative population completed an online questionnaire, providing, among others, insight into their sociodemographics and HRQoL outcomes in 2017. For the current study, a normative population without any cancer diagnoses was derived from the reference panel of CentERdata, which was age- and sex-matched to the SURVAYA study population at the time of questionnaire completion [29]. To maximize the matching, frequency matching was applied, using sex strata (males and females) and age strata (23–39; 40–49; and 50–60 years). SAS (version 9.3 for Windows; SAS institute Inc., Cary, NC) was used for the matching procedure.

### Measures

#### Health-related quality of life (HRQoL)

HRQoL was self-reported by the participants using the European Organisation for Research and Treatment of Cancer (EORTC) Core Quality of Life Questionnaire (EORTC QLQ-C30) [30]. The EORTC QLQ-C30 has demonstrated strong psychometric properties and is a validated and widely used instrument to measure HRQoL in cancer populations [30, 31]. One global QoL scale (overall health and quality of life) and five functioning scale scores (physical, cognitive, emotional, role, and social functioning) were calculated by converting the raw scores into scale scores by linear transformation according to the official EORTC QLQ-C30 Scoring Manual [31]. Final scores per scale range from 0 to 100: a higher score represents a better level of functioning/QoL.

#### Sociodemographic characteristics

Sex (assigned at birth) and age at questionnaire completion were available from the NCR. Marital status, highest educational level achieved, and living status (living alone or together with someone) were self-reported by the participants.

#### Clinical characteristics

Type of cancer (classified according to the Third International Classification of Diseases for Oncology (ICDO-3)), age at and time since cancer diagnosis, type of primary treatment, and tumor stage (classified according to TNM or Ann Arbor Code) were available from the NCR.

#### Health-related conditions

Health-related conditions were self-reported by the participants using the adapted version of the St. Jude Childhood Cancer Survivorship study questionnaire [32]. If a condition was diagnosed more than six months after the cancer diagnosis, it was considered post-cancer diagnosis, as in line with previous analyses [10]. For the analyses, the variable was categorized as follows: (i) no health-related condition, (ii) one health-related condition, and (iii) two or more health-related conditions.

#### Healthcare use

Healthcare use was self-reported by the participants and assessed by the number of visits to their (i) general practitioner (GP) and (ii) medical specialist (MS) in the past year. For the analyses, the two variables were dichotomised using the respective median split, which led to the following categories: (i) zero to one visit to the GP versus two or more visits to the GP and (ii) zero visits to the MS versus one or more visits to the MS.

#### Positive life outlook

Positive life outlook was self-reported by the participants using the EORTC Survivorship questionnaire (EORTC QLQ-SUV111). Positive life outlook consisted of 4 items (e.g., “Do you feel that your life has more purpose?”) and was calculated according to the official EORTC Scoring Manual. The final scale score ranges from 0 to 100; a higher score represents higher levels of positive life outlook [31].

### Data analysis

Descriptive statistics were applied to characterize the participants: numbers and percentages for categorical data and means and standard deviations (SD) for continuous variables. Chi-square tests (categorical data) and independent samples *t*-tests (continuous data) were performed to compare (1) AYA cancer survivors who were included for further analyses (at least one of the six HRQoL scale scores available on record level) with AYA cancer survivors who were excluded from further analyses (no scale score available on record level) and (2) the AYA cancer survivor population with the age- and sex-matched normative population. As differences in HRQoL may exist between AYAs with different tumor types, sensitivity analyses were performed among the AYA cancer survivors stratified by tumor type (if N > 10) with tumor-specific age- and sex-matched normative populations. In addition to statistically significant differences, clinically relevant differences in scale scores between groups were assessed using the interpretation guidelines of Cocks et al. [33]. Hierarchical (multivariable) linear regression analyses were performed to examine which factors were associated with HRQoL (per scale: one global QoL scale and five functioning scales) among AYA cancer survivors. The hierarchical multivariable linear regression models included the following: (i) sociodemographic variables; (ii) sociodemographic and clinical variables; (iii) sociodemographic and clinical variables, and health-related conditions; (iv) sociodemographic and clinical variables, health-related conditions, and healthcare use; and (v) sociodemographic and clinical variables, health-related conditions, healthcare use, and positive life outlook. Independent variables with *p* < 0.1 in univariable linear regression analyses or with associations previously described in literature were included in the hierarchical multivariable linear regression analyses, in the case of no multicollinearity. The latter was tested with tolerance, variance inflation factor, and variance proportion values. All statistical tests were two-sided, and *p*-values < 0.05 were considered statistically significant. IBM Statistical Package for Social Sciences (SPSS) Statistics version 29.0 (SPSS Inc. Chicago, IL) was used for all statistical analyses.

## Results

In total, 4010 AYA cancer survivors participated in the SURVAYA study. Of them, 265 AYAs had no HRQoL scale score available at all and were thus excluded from further analyses, leaving 3745 AYAs included in the final analyses (Appendix Table 1). The included AYAs did not differ from the excluded AYAs in a statistically significant manner with the exception of having received chemotherapy more often and a diagnosis at the youngest and oldest AYA age ranges (18–24 and 35–39 years, respectively).

AYA cancer survivors were, on average, 32 years old at diagnosis and about 12 years post-cancer diagnosis at the moment of questionnaire completion (Appendix Table 1). Three out of five AYAs were female, the top three tumor types included breast cancer (23.7%), germ cell cancer (17.4%), and lymphoid hematological cancer (14.8%), and most participants were treated with surgery (78.0%) and chemotherapy (56.3%).

### HRQoL of the AYA cancer survivor population compared to the normative population

In total, 513 unique peers without a cancer diagnosis were age- and sex-matched to the AYAs (Table 1). AYAs were significantly more likely to have a partner, a university degree, and to cohabitate in comparison with the normative population.

**Table 1:** Sociodemographic characteristics of AYA cancer survivors compared to age- and sex-matched normative population

		AYA cancer survivors		Normative population		*p*-value
		*N* = 3745 (88.0%)		*N* = 513 (12.0%)		
		*N*	%	*N*	%	
Age at questionnaire completion	23–39	1012	27.0	138	26.9	0.992
	40–49	1786	47.7	244	47.6	
	50–60	947	25.3	131	25.5	
	Mean (SD) in years	44.5	7.5	44.4	8.5	
Sex	Male	1457	38.9	200	39.0	0.972
	Female	2288	61.1	313	61.0	
Partner status at time of questionnaire	No partner	619	16.5	250	48.7	< 0.001**
	Partner	3113	83.1	263	51.3	
	Missing	13	0.3	0	0.0	
Education (achieved)	No education or primary education	24	0.6	19	3.7	< 0.001**
	Secondary education	251	6.7	122	23.8	
	Secondary vocational education	1357	36.2	170	33.1	
	Higher (vocational) education	1276	34.1	129	25.1	
	University education	829	22.1	72	14.0	
	Missing	8	0.2	1	0.2	
Living status	Living together^1^	3269	87.3	380	74.1	< 0.001**
	Living alone	468	12.5	133	25.9	
	Missing	8	0.2	0	0.0	

*p < 0.05; **p < 0.01; *N*, number; *SD*, standard deviation; ^1^includes living with partner, parents, children, roommates, or others

All five functioning scales showed statistically significant differences between AYA cancer survivors and the normative population, with AYAs scoring worse (Table 2, Appendix Fig. 1): the differences in physical functioning (91.5 vs. 94.3, *p* < 0.001) and emotional functioning (79.5 vs. 83.4, *p* < 0.001) were trivial; for role functioning (83.2 vs. 91.3, *p* < 0.001) and social functioning (87.9 vs. 93.6, *p* < 0.001), they were small; and for cognitive functioning (77.9 vs. 92.2, *p* < 0.001), the difference was considered large. No significant difference was observed on Global QoL.

**Table 2:** Mean QoL scores per scale of AYA cancer survivors compared to matched normative population

	AYA cancer survivors *N* = 3745 (88%)		Normative population *N* = 513 (12%)		Mean difference (95% CI of the difference)	Clinically relevant difference***	*p*-value
**Global quality of life**							
* N* (observed)	3725		513				
Mean (SD)	75.2 (17.5)		75.7 (18.4)		− 0.5 (− 2.1; 1.1)	Trivial	0.589
Range (observed)	0–100		0 − 100				
**Physical functioning**							
* N* (observed)	3743		513				
Mean (SD)	91.5 (14.0)		94.3 (11.7)		− 2.8 (− 4.0; − 1.8)	Trivial	< 0.001**
Range (observed)	0–100		20 − 100				
**Cognitive functioning**							
* N* (observed)	3731		513				
Mean (SD)	77.9 (24.5)		92.2 (15.8)		− 14.3 (− 15.8; − 12.7)	Large	< 0.001**
Range (observed)	0–100		0 − 100				
**Emotional functioning**							
* N* (observed)	3734		513				
Mean (SD)	79.5 (20.6)		83.4 (20.7)		− 3.9 (− 5.8; − 2.0)	Trivial	< 0.001**
Range (observed)	0–100		0 − 100				
**Role functioning**							
* N* (observed)	3730		513				
Mean (SD)	83.2 (25.4)		91.3 (19.5)		− 8.1 (− 9.9; − 6.2)	Small	< 0.001**
Range (observed)	0–100		0 − 100				
**Social functioning**							
* N* (observed)	3726		513				
Mean (SD)	87.9 (22.1)		93.6 (16.4)		− 5.7 (− 7.3; − 4.1)	Small	< 0.001**
Range (observed)	0–100		0–100				

*p < 0.05; **p < 0.01; ***based on Cocks et al. (2011); *N*, number; *SD*, standard deviation; *CI*, confidence interval

The results of the sensitivity analyses, to assess differences in HRQoL between the tumor-stratified AYA cancer survivor populations and matched normative populations, are shown in Appendix Fig. 2 A-N and Appendix Table 2. Similar to the comparisons between the overall AYA cancer survivor and normative population, no statistically significant difference was observed on Global QoL among the tumor-specific comparisons, while most functioning scales differed significantly, with the worse scores attributed to the tumor-stratified AYA cancer survivors. Differences in cognitive functioning were most often defined as statistically significant and, from a clinical relevance point of view, as large or medium, followed by role and social functioning.

### Factors associated with HRQoL of the AYA cancer survivors

Hierarchical linear regression analyses were performed to examine which factors (sociodemographic, clinical, health-related conditions, healthcare use and positive life outlook) were associated with the six HRQoL scales, respectively, among AYA cancer survivors (Tables 3, 4, 5, 6, 7, 8). The following section describes the results of model 5 of each hierarchical multiple linear regression analysis, which takes into account the other independent variables in the model (in case of *p* < 0.1 in univariable linear regression analyses or with associations previously described in literature, in case of no multicollinearity).

**Table 3:** Standardized betas of hierarchical multiple linear regression analyses evaluating the associations of sociodemographic and clinical factors, health-related conditions, healthcare use and positive life outlook with global QoL

			**Global QoL (*N* = 3683)**				
			**Model 1 (block 1)**	**Model 2 (block 1 + 2)**	**Model 3 (block 1 + 2 + 3)**	**Model 4 (block 1 + 2 + 3 + 4)**	**Model 5 (block 1 + 2 + 3 + 4 + 5)**
			Standardized beta	Standardized beta	Standardized beta	Standardized beta	Standardized beta
**Block 1: sociodemographic characteristics**							
Age at questionnaire completion^4^ (in years)							
Sex	Male		REF	REF	REF	REF	REF
	Female		− 0.045**	− 0.047*	− 0.022	− 0.002	− 0.012
Partner status at time of questionnaire	No partner		REF	REF	REF	REF	REF
	Partner		0.151**	0.140**	0.130**	0.129**	0.126**
Education (achieved)	No education or primary education		− 0.015	− 0.012	− 0.01	− 0.005	− 0.008
	Secondary education		− 0.053**	− 0.051**	− 0.041*	− 0.04*	− 0.044*
	Secondary vocational education		− 0.082**	− 0.075**	− 0.052*	− 0.044*	− 0.066**
	Higher (vocational) education		− 0.039	− 0.036	− 0.023	− 0.016	− 0.026
	University education		REF	REF	REF	REF	REF
Living status	Living together^1^		REF	REF	REF	REF	REF
	Living alone		− 0.014	− 0.018	− 0.015	− 0.007	− 0.002
**Block 2: clinical characteristics**							
Age at diagnosis (in years)							
Time since diagnosis (in years)				0.046**	0.096**	0.076**	0.077**
Tumor type	Breast			REF	REF	REF	REF
	Head and neck			− 0.011	− 0.009	− 0.015	− 0.013
	Digestive track, other			− 0.005	0.003	0.005	0.004
	Colon and rectal			− 0.031	− 0.024	− 0.021	− 0.02
	Bone and soft tissue			− 0.005	− 0.006	− 0.011	− 0.013
	Respiratory tract			− 0.032	− 0.029	− 0.026	− 0.024
	Melanoma			− 0.007	− 0.009	− 0.012	− 0.007
	Other			0.008	0.012	0.01	0.012
	Germ cell			− 0.043	− 0.043	− 0.054	− 0.054
	Female genitalia			− 0.043	− 0.033	− 0.037	− 0.037
	Male genitalia			0.006	0.006	0.007	0.003
	Urinary tract			− 0.039*	− 0.033	− 0.033	− 0.033*
	Lymphoid hematological			− 0.022	− 0.026	− 0.024	− 0.027
	Myeloid hematological			− 0.049	− 0.051	− 0.05	− 0.05
	Thyroid gland			− 0.075**	− 0.067**	− 0.058**	− 0.053**
	Central nervous system			− 0.045	− 0.052*	− 0.054*	− 0.048*
Primary treatment^2^	Chemotherapy	No		REF	REF	REF	REF
		Yes		0.002	0.008	0.001	− 0.004
	Radiotherapy	No		REF	REF	REF	REF
		Yes		− 0.041*	− 0.027	− 0.022	− 0.026
	Hormonal therapy	No		REF	REF	REF	REF
		Yes		− 0.048*	− 0.036	− 0.031	− 0.027
	Targeted therapy	No					
		Yes					
	Surgery	No		REF	REF	REF	REF
		Yes		0.057	0.022	0.021	0.016
	Stem cell therapy	No					
		Yes					
Tumor stage^5^	I			REF	REF	REF	REF
	II			− 0.057**	− 0.055**	− 0.051*	− 0.052**
	III			− 0.056**	− 0.056**	− 0.051**	− 0.056**
	IV			− 0.011	− 0.006	− 0.003	− 0.004
**Block 3: health-related conditions**							
Health-related conditions	No health-related conditions				REF	REF	REF
	1 health-related condition				− 0.101**	− 0.078**	− 0.077**
	2 or more health-related conditions				− 0.268**	− 0.215**	− 0.217**
**Block 4: healthcare use**							
GP visits^3^	0 or 1 GP visit					REF	REF
	2 or more GP visits					− 0.156**	− 0.158**
MS visits^3^	0 MS visits					REF	REF
	1 or more MS visits					− 0.058**	− 0.056**
**Block 5: positive life outlook**							
Positive life outlook	Range 0–100						0.129**
**Model characteristics**							
*R*^2^			0.033	0.053	0.107	0.135	0.151
Adjusted *R*^2^			0.032	0.045	0.099	0.127	0.142
Change in *R*^2^			0.033	0.02	0.054	0.028	0.016
*F*-ratio			18,185**	6608**	13,258**	16,246**	17,977**

*REF*, reference category; *GP*, general practitioner; *MS*, medical specialist; *p < 0.05; **p < 0.01; ^1^includes living with partner, parents, children, roommates, or others; ^2^>100% as participants may have received multiple treatments; ^3^based on median split; ^4^age at questionnaire completion was excluded due to signs of multicollinearity; ^5^missings were categorized (due to high numbers) and included in the analyses but were not interpreted.

**Table 4:** Standardized betas of hierarchical multiple linear regression analyses evaluating the associations of sociodemographic and clinical factors, health-related conditions, healthcare use and positive life outlook with physical functioning

			**Physical functioning (*N* = 3692)**				
			**Model 1 (block 1)**	**Model 2 (block 1 + 2)**	**Model 3 (block 1 + 2 + 3)**	**Model 4 (block 1 + 2 + 3 + 4)**	**Model 5 (block 1 + 2 + 3 + 4 + 5)**
			Standardized beta	Standardized beta	Standardized beta	Standardized beta	Standardized beta
**Block 1: sociodemographic characteristics**							
Age at questionnaire completion^4^ (in years)							
Sex	Male		REF	REF	REF	REF	REF
	Female		− 0.140**	− 0.131**	− 0.109**	− 0.091**	− 0.09**
Partner status at time of questionnaire	No partner		REF	REF	REF	REF	REF
	Partner		0.140**	0.125**	0.118**	0.118**	0.118**
Education (achieved)	No education or primary education		− 0.128**	− 0.122**	− 0.122**	− 0.118**	− 0.118**
	Secondary education		− 0.138**	− 0.124**	− 0.112**	− 0.114**	− 0.113**
	Secondary vocational education		− 0.198**	− 0.175**	− 0.153**	− 0.146**	− 0.145**
	Higher (vocational) education		− 0.083**	− 0.071**	− 0.057**	− 0.051**	− 0.05**
	University education		REF	REF	REF	REF	REF
Living status	Living together^1^		REF	REF	REF	REF	REF
	Living alone		0.005	− 0.003	0.003	0.01	0.01
**Block 2: clinical characteristics**							
Age at diagnosis (in years)				− 0.097**	− 0.085**	− 0.089**	− 0.089**
Time since diagnosis (in years)							
Tumor type	Breast			REF	REF	REF	REF
	Head and neck			− 0.014	− 0.009	− 0.019	− 0.019
	Digestive track, other			− 0.028	− 0.02	− 0.021	− 0.021
	Colon and rectal			− 0.022	− 0.016	− 0.016	− 0.016
	Bone and soft tissue			− 0.11**	− 0.108**	− 0.112**	− 0.112**
	Respiratory tract			− 0.073**	− 0.071**	− 0.069**	− 0.069**
	Melanoma			− 0.023	− 0.023	− 0.027	− 0.027
	Other			0	0.002	0.001	0.001
	Germ cell			− 0.035	− 0.026	− 0.043	− 0.043
	Female genitalia			− 0.036	− 0.027	− 0.036	− 0.036
	Male genitalia			0.005	0.006	0.007	0.007
	Urinary tract			− 0.038*	− 0.033*	− 0.032*	− 0.032*
	Lymphoid hematological			− 0.05	− 0.05	− 0.054	− 0.054
	Myeloid hematological			− 0.075**	− 0.075**	− 0.078**	− 0.078**
	Thyroid gland			− 0.075**	− 0.068**	− 0.059**	− 0.059**
	Central nervous system			− 0.069**	− 0.074**	− 0.073**	− 0.073**
Primary treatment^2^	Chemotherapy	No		REF	REF	REF	REF
		Yes		− 0.024	− 0.023	− 0.028	− 0.027
	Radiotherapy	No		REF	REF	REF	REF
		Yes		− 0.062**	− 0.049**	− 0.044*	− 0.043*
	Hormonal therapy	No		REF	REF	REF	REF
		Yes		− 0.048*	− 0.038	− 0.033	− 0.033
	Targeted therapy	No					
		Yes					
	Surgery	No		REF	REF	REF	REF
		Yes		0	− 0.03	− 0.037	− 0.037
	Stem cell therapy	No		REF	REF	REF	REF
		Yes		− 0.039*	− 0.024	− 0.02	− 0.02
Tumor stage^5^	I			REF	REF	REF	REF
	II			− 0.066**	− 0.062**	− 0.056**	− 0.056**
	III			− 0.083**	− 0.083**	− 0.076**	− 0.076**
	IV			− 0.062**	− 0.059**	− 0.053**	− 0.053**
**Block 3: health-related conditions**							
Health-related conditions	No health-related conditions				REF	REF	REF
	1 health-related condition				− 0.062**	− 0.043**	− 0.043**
	2 or more health-related conditions				− 0.231**	− 0.189**	− 0.188**
**Block 4: healthcare use**							
GP visits^3^	0 or 1 GP visit					REF	REF
	2 or more GP visits					− 0.107**	− 0.106**
MS visits^3^	0 MS visits					REF	REF
	1 or more MS visits					− 0.101**	− 0.101**
**Block 5: positive life outlook**							
Positive life outlook	Range 0–100						− 0.009
**Model characteristics**							
*R*^2^			0.084	0.13	0.172	0.196	0.196
Adjusted *R*^2^			0.083	0.122	0.165	0.188	0.188
Change in *R*^2^			0.084	0.045	0.043	0.023	0
*F*-ratio			48,548**	17,054**	22,401**	24,729**	24,066**

**Table 5:** Standardized betas of hierarchical multiple linear regression analyses evaluating the associations of sociodemographic and clinical factors, health-related conditions, healthcare use and positive life outlook with role functioning

			**Role functioning (*N* = 3689)**				
			**Model 1 (block 1)**	**Model 2 (block 1 + 2)**	**Model 3 (block 1 + 2 + 3)**	**Model 4 (block 1 + 2 + 3 + 4)**	**Model 5 (block 1 + 2 + 3 + 4 + 5)**
			Standardized beta	Standardized beta	Standardized beta	Standardized beta	Standardized beta
**Block 1: sociodemographic characteristics**							
Age at questionnaire completion^4^ (in years)							
Sex	Male		REF	REF	REF	REF	REF
	Female		− 0.113**	− 0.092**	− 0.069**	− 0.047*	− 0.046*
Partner status at time of questionnaire	No partner		REF	REF	REF	REF	REF
	Partner		0.127**	**0.114	0.105**	0.104**	0.104**
Education (achieved)	No education or primary education		− 0.064**	− 0.059**	− 0.059**	− 0.054**	− 0.054**
	Secondary education		− 0.072**	− 0.058**	− 0.046**	− 0.047**	− 0.046**
	Secondary vocational education		− 0.138**	− 0.116**	− 0.094**	− 0.086**	− 0.084**
	Higher (vocational) education		− 0.086**	− 0.074**	− 0.06**	− 0.053**	− 0.052**
	University education		REF	REF	REF	REF	REF
Living status	Living together^1^		REF	REF	REF	REF	REF
	Living alone		− 0.024	− 0.029	− 0.023	− 0.014	− 0.014
**Block 2: clinical characteristics**							
Age at diagnosis (in years)				− 0.072**	− 0.06**	− 0.063**	− 0.064**
Time since diagnosis (in years)							
Tumor type	Breast			REF	REF	REF	REF
	Head and neck			0.01	0.015	0.005	0.004
	Digestive track, other			− 0.008	0	0	0
	Colon and rectal			− 0.01	− 0.005	− 0.003	− 0.003
	Bone and soft tissue			− 0.042*	− 0.039*	− 0.045*	− 0.045*
	Respiratory tract			− 0.043*	− 0.041*	− 0.038*	− 0.038*
	Melanoma			0.023	0.023	0.019	0.018
	Other			0.004	0.007	0.005	0.005
	Germ cell			− 0.002	0.007	− 0.011	− 0.011
	Female genitalia			− 0.021	− 0.012	− 0.02	− 0.02
	Male genitalia			0.007	0.008	0.009	0.009
	Urinary tract			− 0.029	− 0.023	− 0.022	− 0.022
	Lymphoid hematological			0.013	0.013	0.009	0.009
	Myeloid hematological			− 0.034	− 0.034	− 0.037	− 0.037
	Thyroid gland			− 0.063**	− 0.056**	− 0.045*	− 0.046*
	Central nervous system			− 0.086**	− 0.091**	− 0.091**	− 0.091**
Primary treatment^2^	Chemotherapy	No		REF	REF	REF	REF
		Yes		− 0.03	− 0.029	− 0.034	− 0.034
	Radiotherapy	No		REF	REF	REF	REF
		Yes		− 0.045*	− 0.031	− 0.025	− 0.025
	Hormonal therapy	No		REF	REF	REF	REF
		Yes		− 0.041	− 0.03	− 0.024	− 0.024
	Targeted therapy	No					
		Yes					
	Surgery	No		REF	REF	REF	REF
		Yes		0.069	0.037	0.03	0.031
	Stem cell therapy	No		REF	REF	REF	REF
		Yes		− 0.014	0.002	0.005	0.005
Tumor stage^5^	I			REF	REF	REF	REF
	II			− 0.047*	− 0.043*	− 0.037	− 0.037
	III			− 0.055**	− 0.056**	− 0.049*	− 0.048*
	IV			− 0.051**	− 0.048**	− 0.043*	− 0.043*
**Block 3: health-related conditions**							
Health-related conditions	No health-related conditions				REF	REF	REF
	1 health-related condition				− 0.051**	− 0.028	− 0.028
	2 or more health-related conditions				− 0.24**	− 0.187**	− 0.186**
**Block 4: healthcare use**							
GP visits^3^	0 or 1 GP visit					REF	REF
	2 or more GP visits					− 0.150**	− 0.15**
MS visits^3^	0 MS visits					REF	REF
	1 or more MS visits					− 0.094**	− 0.094**
**Block 5: positive life outlook**							
Positive life outlook	Range 0–100						− 0.01
**Model characteristics**							
*R*^2^			0.049	0.087	0.134	0.167	0.167
Adjusted *R*^2^			0.047	0.079	0.126	0.159	0.159
Change in *R*^2^			0.049	0.038	0.047	0.034	0
*F*-ratio			27,152**	10,866**	16,585**	20,378**	19,837**

*REF*, reference category; *GP*, general practitioner; *MS*, medical specialist; *p < 0.05; **p < 0.01; ^1^includes living with partner, parents, children, roommates, or others; ^2^>100% as participants may have received multiple treatments; ^3^based on median split; ^4^age at questionnaire completion was excluded due to signs of multicollinearity; ^5^missings were categorized (due to high numbers) and included in the analyses, but not interpreted.

**Table 6:** Standardized betas of hierarchical multiple linear regression analyses evaluating the associations of sociodemographic and clinical factors, health-related conditions, healthcare use and positive life outlook with emotional functioning

			**Emotional functioning (N = 3689)**				
			**Model 1 (block 1)**	**Model 2 (block 1 + 2)**	**Model 3 (block 1 + 2 + 3)**	**Model 4 (block 1 + 2 + 3 + 4)**	**Model 5 (block 1 + 2 + 3 + 4 + 5)**
			Standardized beta	Standardized beta	Standardized beta	Standardized beta	Standardized beta
**Block 1: sociodemographic characteristics**							
Age at questionnaire completion^4^ (in years)							
Sex	Male		REF	REF	REF	REF	REF
	Female		− 0.110**	− 0.096**	− 0.074**	− 0.054*	− 0.059*
Partner status at time of questionnaire	No partner		REF	REF	REF	REF	REF
	Partner		0.104**	0.097**	0.088**	0.084**	0.083**
Education (achieved)	No education or primary education		− 0.021	− 0.02	− 0.018	− 0.012	− 0.013
	Secondary education		− 0.011	− 0.015	− 0.006	− 0.003	− 0.005
	Secondary vocational education		− 0.035	− 0.036	− 0.016	− 0.007	− 0.018
	Higher (vocational) education		0.007	0.007	0.019	0.025	0.02
	University education		REF	REF	REF	REF	REF
Living status	Living together^1^		REF	REF	REF	REF	REF
	Living alone		0	− 0.003	− 0.001	0.005	0.008
**Block 2: clinical characteristics**							
Age at diagnosis (in years)							
Time since diagnosis (in years)				0.066**	0.109**	0.094**	0.095**
Tumor type	Breast			REF	REF	REF	REF
	Head and neck			0.017	0.024	0.019	0.021
	Digestive track, other			− 0.008	0	0.004	0.003
	Colon and rectal			− 0.007	0	0.005	0.005
	Bone and soft tissue			0.007	0.008	0.002	0.001
	Respiratory tract			− 0.01	− 0.005	− 0.003	− 0.001
	Melanoma			0.052*	0.052*	0.049*	0.051*
	Other			− 0.009	− 0.004	− 0.006	− 0.005
	Germ cell			0.023	0.024	0.015	0.015
	Female genitalia			0.024	0.036	0.034	0.035
	Male genitalia			0.01	0.011	0.011	0.009
	Urinary tract			− 0.016	− 0.009	− 0.008	− 0.008
	Lymphoid hematological			− 0.002	0.022	0.025	0.025
	Myeloid hematological			0.007	0.026	0.03	0.029
	Thyroid gland			− 0.026	− 0.018	− 0.011	− 0.008
	Central nervous system			− 0.045*	− 0.043*	− 0.045*	− 0.044*
Primary treatment^2^	Chemotherapy	No		REF	REF	REF	REF
		Yes		− 0.023	− 0.011	− 0.017	− 0.02
	Radiotherapy	No		REF	REF	REF	REF
		Yes		− 0.017	− 0.002	0	− 0.001
	Hormonal therapy	No		REF	REF	REF	REF
		Yes		− 0.012	− 0.002	0.003	0.006
	Targeted therapy	No		REF	REF	REF	REF
		Yes		− 0.01	− 0.014	− 0.017	− 0.017
	Surgery	No					
		Yes					
	Stem cell therapy	No					
		Yes					
Tumor stage^5^	I						
	II						
	III						
	IV						
**Block 3: health-related conditions**							
Health-related conditions	No health-related conditions				REF	REF	REF
	1 health-related condition				− 0.097**	− 0.073**	− 0.073**
	2 or more health-related conditions				− 0.233**	− 0.178**	− 0.179**
**Block 4: healthcare use**							
GP visits^3^	0 or 1 GP visit					REF	REF
	2 or more GP visits					− 0.188**	− 0.19**
MS visits^3^	0 MS visits					REF	REF
	1 or more MS visits					− 0.018	− 0.017
**Block 5: positive life outlook**							
Positive life outlook	Range 0–100						0.064**
**Model characteristics**							
*R*^2^			0.025	0.041	0.082	0.115	0.119
Adjusted *R*^2^			0.023	0.034	0.075	0.108	0.112
Change in *R*^2^			0.025	0.016	0.041	0.033	0.004
*F*-ratio			13,472**	5829**	11,265**	15,385**	15,482**

*REF*, reference category; *GP*, general practitioner; *MS*, medical specialist; *p < 0.05; **p < 0.01; ^1^includes living with partner, parents, children, roommates, or others; ^2^>100% as participants may have received multiple treatments; ^3^based on median split; ^4^age at questionnaire completion was excluded due to signs of multicollinearity; ^5^missings were categorized (due to high numbers) and included in the analyses, but not interpreted.

**Table 7:** Standardized betas of hierarchical multiple linear regression analyses evaluating the associations of sociodemographic and clinical factors, health-related conditions, healthcare use and positive life outlook with cognitive functioning

			**Cognitive functioning (N = 3686)**				
			**Model 1 (block 1)**	**Model 2 (block 1 + 2)**	**Model 3 (block 1 + 2 + 3)**	**Model 4 (block 1 + 2 + 3 + 4)**	**Model 5 (block 1 + 2 + 3 + 4 + 5)**
			Standardized beta	Standardized beta	Standardized beta	Standardized beta	Standardized beta
**Block 1: sociodemographic characteristics**							
Age at questionnaire completion^4^ (in years)							
Sex	Male		REF	REF	REF	REF	REF
	Female		− 0.147**	− 0.135**	− 0.112**	− 0.095**	− 0.095**
Partner status at time of questionnaire	No partner		REF	REF	REF	REF	REF
	Partner		0.072**	0.063**	0.053**	0.051*	0.052*
Education (achieved)	No education or primary education		− 0.024	− 0.029	− 0.027	− 0.023	− 0.023
	Secondary education		− 0.109**	− 0.106**	− 0.098**	− 0.095**	− 0.095**
	Secondary vocational education		− 0.162**	− 0.156**	− 0.136**	− 0.129**	− 0.127**
	Higher (vocational) education		− 0.086**	− 0.078**	− 0.066**	− 0.061**	− 0.06**
	University education		REF	REF	REF	REF	REF
Living status	Living together^1^		REF	REF	REF	REF	REF
	Living alone		− 0.007	− 0.008	− 0.005	0.001	0.001
**Block 2: clinical characteristics**							
Age at diagnosis (in years)				− 0.028	− 0.015	− 0.017	− 0.018
Time since diagnosis (in years)				0.076**	0.12**	0.106**	0.106**
Tumor type	Breast			REF	REF	REF	REF
	Head and neck			0.035	0.039*	0.033	0.033
	Digestive track. other			0.013	0.021	0.022	0.022
	Colon and rectal			0.028	0.034*	0.037*	0.037*
	Bone and soft tissue			0.026	0.027	0.022	0.022
	Respiratory tract			0.016	0.019	0.021	0.021
	Melanoma			0.091**	0.09**	0.087**	0.086**
	Other			− 0.005	− 0.002	− 0.004	− 0.004
	Germ cell			0.015	0.019	0.01	0.01
	Female genitalia			0.033	0.042	0.038	0.038
	Male genitalia			0.017	0.017	0.017	0.017
	Urinary tract			0.004	0.01	0.01	0.01
	Lymphoid hematological			− 0.024	− 0.024	− 0.023	− 0.023
	Myeloid hematological			− 0.01	− 0.01	− 0.008	− 0.008
	Thyroid gland			− 0.026	− 0.016	− 0.011	− 0.011
	Central nervous system			− 0.089**	− 0.093**	− 0.096**	− 0.097**
Primary treatment^2^	Chemotherapy	No		REF	REF	REF	REF
		Yes		− 0.082**	− 0.075**	− 0.081**	− 0.08**
	Radiotherapy	No		REF	REF	REF	REF
		Yes		− 0.024	− 0.012	− 0.009	− 0.008
	Hormonal therapy	No		REF	REF	REF	REF
		Yes		− 0.028	− 0.017	− 0.013	− 0.013
	Targeted therapy	No		REF	REF	REF	REF
		Yes		0.038*	0.033	0.031	0.031
	Surgery	No		REF	REF	REF	REF
		Yes		0.006	− 0.026	− 0.027	− 0.027
	Stem cell therapy	No					
		Yes					
Tumor stage^5^	I			REF	REF	REF	REF
	II			− 0.023	− 0.021	− 0.018	− 0.018
	III			− 0.024	− 0.023	− 0.02	− 0.02
	IV			− 0.028	− 0.024	− 0.021	− 0.021
**Block 3: health-related conditions**							
Health-related conditions	No health-related conditions				REF	REF	REF
	1 health-related condition				− 0.086**	− 0.066**	− 0.066**
	2 or more health-related conditions				− 0.244**	− 0.200**	− 0.199**
**Block 4: healthcare use**							
GP visits^3^	0 or 1 GP visit					REF	REF
	2 or more GP visits					− 0.141**	− 0.141**
MS visits^3^	0 MS visits					REF	REF
	1 or more MS visits					− 0.031	− 0.031
**Block 5: positive life outlook**							
Positive life outlook	Range 0 − 100						− 0.009
**Model characteristics**							
*R*^2^			0.048	0.098	0.142	0.162	0.162
Adjusted *R*^2^			0.046	0.089	0.134	0.154	0.154
Change in *R*^2^			0.048	0.05	0.045	0.020	0
*F*-ratio			26,428**	11,966**	17,286**	19,118**	1862**

*REF*, reference category; *GP*, general practitioner; *MS*, medical specialist; *p < 0.05; **p < 0.01; ^1^includes living with partner, parents, children, roommates, or others; ^2^>100% as participants may have received multiple treatments; ^3^based on median split; ^4^age at questionnaire completion was excluded due to signs of multicollinearity; ^5^missings were categorized (due to high numbers) and included in the analyses but not interpreted.

**Table 8:** Standardized betas of hierarchical multiple linear regression analyses evaluating the associations of sociodemographic and clinical factors, health-related conditions, healthcare use and positive life outlook with social functioning

			Social functioning ***(N= 3689)***				
			**Model 1 (block 1)**	**Model 2 (block 1 + 2)**	**Model 3 (block 1 + 2 + 3)**	**Model 4 (block 1 + 2 + 3 + 4)**	**Model 5 (block 1 + 2 + 3 + 4 + 5)**
			Standardized beta	Standardized beta	Standardized beta	Standardized beta	Standardized beta
**Block 1: sociodemographic characteristics**							
Age at questionnaire completion^4^ (in years)							
Sex	Male		REF	REF	REF	REF	REF
	Female		− 0.115**	− 0.084**	− 0.063**	− 0.046*	− 0.047*
Partner status at time of questionnaire	No partner		REF	REF	REF	REF	REF
	Partner		0.148**	0.134**	0.123**	0.123**	0.123**
Education (achieved)	No education or primary education		− 0.048**	− 0.043**	− 0.042**	− 0.039*	− 0.039*
	Secondary education		− 0.073**	− 0.062**	− 0.054**	− 0.052**	− 0.053**
	Secondary vocational education		− 0.076**	− 0.057**	− 0.039	− 0.032	− 0.033
	Higher (vocational) education		− 0.062**	− 0.052*	− 0.04*	− 0.034	− 0.035
	University education		REF	REF	REF	REF	REF
Living status	Living together^1^		REF	REF	REF	REF	REF
	Living alone		0.035	0.03	0.033	0.04*	0.040*
**Block 2: clinical characteristics**							
Age at diagnosis (in years)				− 0.046**	− 0.035*	− 0.038*	− 0.038*
Time since diagnosis (in years)				0.023	0.065**	0.047**	0.047**
Tumor type	Breast			REF	REF	REF	REF
	Head and neck			− 0.01	− 0.005	− 0.012	− 0.012
	Digestive track. other			− 0.019	− 0.012	− 0.012	− 0.012
	Colon and rectal			− 0.016	− 0.01	− 0.008	− 0.008
	Bone and soft tissue			− 0.036	− 0.035	− 0.039*	− 0.039*
	Respiratory tract			− 0.043*	− 0.04*	− 0.039*	− 0.038*
	Melanoma			0.004	0.003	0.001	0.001
	Other			0.01	0.014	0.012	0.012
	Germ cell			0.001	0.006	− 0.004	− 0.004
	Female genitalia			− 0.081**	− 0.073**	− 0.078**	− 0.078**
	Male genitalia			− 0.009	− 0.009	− 0.008	− 0.009
	Urinary tract			− 0.025	− 0.019	− 0.019	− 0.019
	Lymphoid hematological			− 0.016	− 0.02	− 0.021	− 0.021
	Myeloid hematological			− 0.044	− 0.046	− 0.046	− 0.046
	Thyroid gland			− 0.062**	− 0.052*	− 0.046*	− 0.046*
	Central nervous system			− 0.112**	− 0.113**	− 0.115**	− 0.114**
Primary treatment^2^	Chemotherapy	No		REF	REF	REF	REF
		Yes		− 0.041	− 0.037	− 0.043	− 0.043
	Radiotherapy	No		REF	REF	REF	REF
		Yes		− 0.047*	− 0.035	− 0.031	− 0.031
	Hormonal therapy	No		REF	REF	REF	REF
		Yes		− 0.034	− 0.023	− 0.019	− 0.019
	Targeted therapy	No					
		Yes					
	Surgery	No		REF	REF	REF	REF
		Yes		0.042	0.009	0.007	0.006
	Stem cell therapy	No		REF	REF	REF	REF
		Yes		− 0.013	0.003	0.004	0.004
Tumor stage^5^	I			REF	REF	REF	REF
	II			− 0.06**	− 0.056**	− 0.052**	− 0.052**
	III			− 0.06**	− 0.06**	− 0.055**	− 0.055**
	IV			− 0.058**	− 0.053**	− 0.05**	− 0.05**
**Block 3: health-related conditions**							
Health-related conditions	No health-related conditions				REF	REF	REF
	1 health-related condition				− 0.052**	− 0.033	− 0.033
	2 or more health-related conditions				− 0.231**	− 0.188**	− 0.188**
**Block 4: healthcare use**							
GP visits^3^	0 or 1 GP visit					REF	REF
	2 or more GP visits					− 0.118**	− 0.118**
MS visits^3^	0 MS visits					REF	REF
	1 or more MS visits					− 0.058**	− 0.057**
**Block 5: positive life outlook**							
Positive life outlook	Range 0 − 100						0.005
**Model characteristics**							
*R*^2^			0.039	0.075	0.116	0.134	0.134
Adjusted *R*^2^			0.037	0.066	0.108	0.125	0.125
Change in *R*^2^			0.039	0.036	0.042	0.018	0
*F*-ratio			21,083**	8937**	13,748**	15,299**	14,895**

*REF*, reference category; *GP*, general practitioner; *MS*, medical specialist; *p < 0.05; **p < 0.01; ^1^includes living with partner, parents, children, roommates, or others; ^2^>100% as participants may have received multiple treatments; ^3^Based on median split; ^4^age at questionnaire completion was excluded due to signs of multicollinearity; ^5^missings were categorized (due to high numbers) and included in the analyses, but not interpreted.

Being female was negatively associated with all five functioning scales. No education to higher (vocational) education was negatively associated with global QoL and all functioning scales, except emotional functioning, although not all were statistically significant. Having a partner was positively associated with global QoL and physical, role, cognitive, and social functioning. Living alone was positively associated with social functioning.

Looking at the clinical variables, results showed that older age at diagnosis was negatively associated with physical, role, and social functioning. A longer time since diagnosis was positively associated with global QoL and emotional, cognitive, and social functioning. The following tumor-specific results were all found in comparison to the reference group (breast cancer). A sarcoma diagnosis and a cancer diagnosis of the respiratory tract were both negatively associated with physical, role, and social functioning. A cancer diagnosis of the female genitalia was negatively associated with social functioning. A cancer diagnosis of the urinary tract was negatively associated with global QoL and physical functioning. Being diagnosed with a myeloid hematological malignancy was negatively associated with physical functioning. A cancer diagnosis of the thyroid gland was negatively associated with global QoL and physical, role, and social functioning. A cancer diagnosis of the central nervous system was negatively associated with global QoL and all five functioning scales. A colorectal cancer diagnosis was positively associated with cognitive functioning. A melanoma diagnosis was positively associated with emotional and cognitive functioning. Regarding treatment, results showed that having received chemotherapy was negatively associated with cognitive functioning, while having received radiotherapy was negatively associated with physical functioning, compared to not receiving the treatment respectively. Tumor stages II to IV were all negatively associated with physical and social functioning. In addition, stage II was negatively associated with global QoL, stage III with global QoL and role functioning, and stage IV was negatively associated with role functioning. Stage I was considered as reference.

Having one health-related condition was negatively associated with global QoL and physical, emotional, and cognitive functioning, while having two or more health-related conditions was negatively associated with all six scales (reference group is no health-related conditions). Visiting the GP two times or more per year was negatively associated with global QoL and all five functioning scales (reference is no or one GP visit per year). Visiting the MS once or more per year was negatively associated with global QoL and physical, role, and social functioning (reference is no MS visits).

An increased positive life outlook was positively associated with global QoL and emotional functioning.

## Discussion

This study shows the significant impact of a cancer diagnosis and subsequent treatment on long-term HRQoL to the detriment of AYAs compared to an age- and sex-matched normative population. Although no significant difference was found on global QoL, all five functioning scores of AYAs were significantly worse in comparison to the normative population, with clinical differences ranging from trivial to large. Fairly similar trends were seen from a tumor-specific perspective. When examining associated factors, being female, lower educational attainment, older age at diagnosis, tumor type, chemotherapy, radiotherapy, advanced tumor stages, having one or more health-related conditions, and seeking healthcare services were negatively associated with several to all HRQoL scales in comparison with their reference groups.

Our study is partly comparable to two other HRQoL studies performed among long-term AYA cancer survivors internationally. A Canadian and an American study observed similar trends, showing worse (physical and mental) HRQoL among long-term AYA cancer survivors in comparison to controls [11, 12]. However, no exact comparisons can be made due to the use of different HRQoL measures [11, 12, 34]. With the exception of physical and mental health, no other HRQoL domains examined in the SURVAYA study are available in these studies, particularly emotional, role, cognitive, and social functioning. As such, the availability of these domains in our study via the EORTC QLQ-C30 provides the opportunity to have a more in-depth look at the type of HRQoL impact. In addition, mental health is not directly comparable to one of the EORTC QLQ-C30 scores, as this score is covered by both emotional and cognitive functioning. The sensitivity analyses showed cognitive functioning to be statistically significantly different between all tumor-stratified AYA populations and matched normative populations (except for melanoma). In addition, differences in cognitive functioning were the largest compared to the other functioning scales. Although not stratified on tumor type, previous research has shown that AYAs were more likely to experience cognitive dysfunction compared to those without cancer and that cognitive dysfunction was associated with psychological distress [35]. It is likely that diminished cognitive functioning has a broad impact on daily life, such as education and employment, health behaviors, depression and anxiety, and romantic and social relationships among others [36]. As cognition develops at different points in life, it is important to have a closer look at when diagnosis and treatment take place and how this affects cognitive functioning over time. Similar to our results, previous studies found no significant difference in global QoL among AYAs and the normative population. This could be due to experiencing post-traumatic growth, or it could be that the global QoL score is too generic to capture the unique, age-specific issues with which AYAs are dealing, while these might be better represented in the functioning scales [14, 37, 38].

Looking at the factors associated with HRQoL, several subgroups at risk can be identified, of which some are highlighted below. Firstly, the female sex was associated with worse functioning (all scales) compared to males, which is in line with previous research [14, 39]. Geue et al. hypothesized that differences in QoL among sexes could be caused by differences in treatment (such as dose and length) or the expression of emotions [39]. In line with this, in our study, females underwent systemic treatment more often such as chemotherapy, radiotherapy, and hormonal therapy, compared to males (data not shown). However, as our study is limited by the lack of detailed treatment information regarding dose and length, future research should take a closer look at this. Regarding the expression of emotions, this is explained by gendered norms whereby women are socialized to express their emotions, while men are socialized to downplay theirs. This might lead to different healthcare needs and healthcare use between sexes [40, 41]. In 2023, the Comprehensive Cancer Organization Netherlands published a report highlighting differences in outcomes, including diagnosis, treatment, survival, quality of life, and clinical trial participation between male and female patients [42]. They highlight that, although personalized oncology care has received increased attention, differences in outcomes between sexes have remained underexposed. As male survivors are underrepresented in our study, it limits the generalizability of our results. Future research should focus on improved recruitment strategies and strive for equal representation of subgroups to provide input for the development of risk-stratified survivorship care.

Our results showed that having a partner was positively associated with all HRQoL outcomes; however, inconsistent results have been found in other studies. Leung et al. looked specifically into the long-term HRQoL between breast cancer survivors (not AYA-specific) with and without a partner [43]. Their results showed poorer HRQoL (physical and mental) for those without partners compared to those with partners, which aligns with our results. They explained this by the mediating effect of social support: patients’ partners provide social support which helps them to maintain well-being and HRQoL. The provided social support may cover a range of domains, including emotional, informational, and practical, for example [7, 43]. In contrast, the AYA-specific study of Schulte et al. found no significant association between physical functioning and partner status and an inverse significant association between mental health and having a partner [12]. They showed that a higher perceived social support was related to worse physical health. They hypothesized that those with worse physical health are more in need of support. Differences in study results might also be explained by differences in conceptualization. Both study explanations could be applicable, and social support might be especially important to this unique population and their age-specific needs. Future research should focus on unraveling the complex relationship between HRQoL and social support to generate input for future interventions [12].

Lastly, negative associations were found between having one or multiple health-related conditions and all HRQoL scales. In line with this, results showed negative associations between GP visits and MS visits and all HRQoL scales: AYAs who visit the GP and/or MS more often are more likely to have worse HRQoL scores, compared to those who visit their GP/MS less often. These findings are as expected and represent diminished HRQoL among those who experience issues in one or multiple domains, as similarly seen in other studies [15, 18, 24]. Harju et al. showed that late effects were associated with poor mental health among AYAs (16–25 years old at cancer diagnosis) [15]. Similarly, the study of Becktell et al., focusing on pediatric and AYA cancer survivors, indicated worse HRQoL scores for those with more than two late effects, compared to those with up to two late effects [18]. In the study of Kirchhoff et al., AYA cancer survivors with more than one cancer diagnosis reported worse QoL scores compared to their reference group [11]. Our study confirms that these health-related conditions, which are common effects of cancer (treatment), can significantly impact HRQoL. Currently, follow-up care is solely tumor-focused and not age-focused; long-term age-specific survivorship care has not yet been well developed for AYAs. Together, this underlines the need for holistic and age-specific survivorship care, in which health-related conditions are considered. Notably, in this study, we had no insight into the severity of the health-related conditions, and all types of conditions were combined, which might be of interest for future research.

### Implications for healthcare and research

In combination with the literature highlighting the high burden of long-term and late effects among AYA cancer survivors, this study underlines the importance of timely AYA-specific, risk-stratified, long-term survivorship care [7, 12, 44]. It is needed to identify and address factors impacting HRQoL with the goal of minimizing issues or preventing them. Many AYA cancer survivors who participated in this study were diagnosed well before AYA-specific care was officially developed and implemented in the Netherlands [45]. Although, over time, progress has been made, and more and more AYAs are receiving AYA-specific care in the Netherlands, AYA-specific survivorship care, on the other hand, is still often lacking. This is the case in the Netherlands, as well as in many other countries in the world. Which survivorship care model is most optimal for AYA cancer survivors has yet to be determined [3]. Having (inter)national standards/guidelines on what survivorship care should ideally look like might support the realization of it, support different stakeholders in providing and receiving appropriate care, and improve (equal) access to care on a national level regardless of the type of hospital, healthcare provider, or residency. In addition, AYA-specific survivorship care should appreciate the variety of HRQoL domains that can be impacted by cancer (treatment). For this, a multidisciplinary team should be part of standard survivorship care to cover these domains [7, 37]. To illustrate, a psychologist or social worker could be part of this multidisciplinary team to provide tailored support regarding an AYA’s emotional or social functioning respectively. For the future, it would be interesting to have a closer look at which and how specific factors impact HRQoL. To illustrate, the AYA cancer survivors were more likely to have a partner, to have completed secondary vocational education or higher, and to be living together with others instead of alone, which are often considered protective factors. However, the HRQoL functioning scale scores of the AYA cancer survivors were significantly lower in comparison to the matched normative population. This indicates that achieving milestones, which are characteristic of this phase of life, does not necessarily equate to optimal HRQoL. It should be explored how AYA cancer survivors are best supported and, following this, care for AYA cancer survivors should be tailored and adapted accordingly to account for the complexity of survivorship.

Cancer-generic HRQoL measures or those developed for other age populations (i.e., not validated in AYAs) might not adequately measure or detect changes in HRQoL among AYAs [34, 37, 44, 46, 47]. Although the EORTC QLQ-C30 covers more than solely physical and mental HRQoL, as a standalone measure, it does not cover the detailed assessment of AYA-specific domains. Fortunately, both Patient-Reported Outcomes Measurement Information System (PROMIS) and the EORTC Quality of Life Group are leading an American- and European-centered initiative, respectively, to develop AYA-specific HRQoL measurements that cover the full AYA age range and are psychometrically robust [34, 44, 46–50]. The development of survivorship care and new research initiatives should take place in close collaboration and co-creation with different stakeholders, and especially with AYAs themselves [51, 52].

### Strengths and limitations

The strengths of this study are the long-term follow-up time (5–20 years since cancer diagnosis), large sample size (3745 participating AYAs), population-based data, broad coverage of tumor types, use of a validated and widely implemented cancer-specific and HRQoL-specific questionnaire, and the inclusion of an age- and sex-matched normative population.

However, some limitations need to be addressed. Firstly, selection bias may have played a role in both the SURVAYA study and the normative population data collection; some subgroups were less well represented, which might have impacted the results and the generalizability of it [27]. Also, given that the included AYA cancer survivors were significantly more often treated with chemotherapy compared to the excluded AYA cancer survivors (no HRQoL data available) and that chemotherapy was negatively associated with cognitive functioning (regression analyses), it is possible that this overrepresentation may have influenced the results. This treatment-related effect potentially represents the phenomenon of “chemobrain” Secondly, the matching was only based on the variables age and sex. Ideally, more variables were taken into account, such as ethnicity, educational attainment, or socioeconomic status, in order to establish a normative population that is more similar to the AYA cancer survivor population and to have a more objective look at the impact of cancer on the HRQoL among AYAs. Also, data from the normative population was either available in 2017 or 2020, which was either completely before or completely during the COVID-19 pandemic respectively. As the SURVAYA study is conducted partly before and partly during the COVID-19 pandemic, this might have impacted our results. However, as the normative population of 2017 included more young participants due to extra sampling, creating a matched normative population large enough for analyses was more feasible using the 2017 cohort. Thirdly, the NCR provides general primary treatment data, but nothing related to treatment specifics, such as dosages, specific cytostatic agents, or duration, or subsequent treatment(s). Therefore, analyses focusing on treatment-specific effects and associations were limited. Also, due to signs of multicollinearity, the variables age at diagnosis and time since diagnosis were included in the regression analyses, while age at questionnaire completion was excluded. Fourthly, the median split was applied to dichotomize the healthcare use variables; however, this has been questioned as an ideal cut-off method from a methodological point of view. For now, the median split was applied as it was in line with other studies using similar variables and as no golden standard is available yet. Fifthly, the cross-sectional nature of this study prevents us from defining causal relationships. Longitudinal studies focusing on both short- and long-term HRQoL of AYAs and potential risk and protective factors should be conducted [44].

## Conclusion

To conclude, this study showed significantly hampered functioning across all domains—most pronounced in cognitive functioning—among many long-term AYA cancer survivors. This highlights the importance of the development and implementation of interventions and timely evidence-based, risk-stratified survivorship care for current and future AYA cancer survivors.

## Supplementary Information

Below is the link to the electronic supplementary material.

ESM 1 (93.0 KB DOCX)

ESM 2 (1.12 MB DOCX)

ESM 3 (20.8 KB DOCX)

ESM 4 (42.5 KB DOCX)

## Data Availability

The dataset generated and analyzed in this study is available from the corresponding author on reasonable request. The data are not publicly available due to privacy issues.
