# Supplementary material for: Health-related quality of life of long-term adolescent and young adult (AYA) cancer survivors compared to a matched normative population: results of the SURVAYA study
Source: J Cancer Surviv. 2025 Jun 17;20(Suppl 1):628–51. doi: 10.1007/s11764-025-01818-0 (PMC13676661; doi:10.1007/s11764-025-01818-0)
Supplement: Supplementary file 1 [file 11764_2025_1818_MOESM1_ESM.docx]

**Appendix Figure 1. Mean QoL scores per scale of AYA cancer survivors compared to matched normative population**

Note: Scores can range from 0 to 100: a higher score implies better global QoL or functioning; * p<0.05 and trivial clinical difference; ** p<0.05 and small clinical difference; *** p<0.05 and medium clinical difference; **** p<0.05 and large clinical difference.
