# Supplementary material for: Health-related quality of life of long-term adolescent and young adult (AYA) cancer survivors compared to a matched normative population: results of the SURVAYA study
Source: J Cancer Surviv. 2025 Jun 17;20(Suppl 1):628–51. doi: 10.1007/s11764-025-01818-0 (PMC13676661; doi:10.1007/s11764-025-01818-0)
Supplement: Supplementary file 2 [file 11764_2025_1818_MOESM2_ESM.doc]

**Appendix Figure 2A-N.** Mean QoL scores per scale of AYA cancer survivors compared to matched normative population, per tumor type

a.	b.

c.	d.

e.	f.

g.	h.

i.	j.

k.	l.

m.	n.

Note: Scores can range from 0 to 100: a higher score implies better global QoL or functioning; * p<0.05 and trivial clinical difference; ** p<0.05 and small clinical difference; *** p<0.05 and medium clinical difference; **** p<0.05 and large clinical difference.
