# Supplementary material for: Health-related quality of life of long-term adolescent and young adult (AYA) cancer survivors compared to a matched normative population: results of the SURVAYA study
Source: J Cancer Surviv. 2025 Jun 17;20(Suppl 1):628–51. doi: 10.1007/s11764-025-01818-0 (PMC13676661; doi:10.1007/s11764-025-01818-0)
Supplement: Supplementary file 3 [file 11764_2025_1818_MOESM3_ESM.docx]

**Appendix Table 1. Sociodemographic and clinical characteristics of AYA cancer survivors, stratified by inclusion versus exclusion**

|  |  |  | All survivors | | Included AYAs | | Excluded AYAs | |  |
| --- | --- | --- | --- | --- | --- | --- | --- | --- | --- |
|  |  |  | N= 4010 (100%) | | N= 3745 (93,4%) | | N= 265 (6,6%) | |  |
|  |  |  | N | % | N | % | N | % | p-value |
| Age at diagnosis | 18-24 years |  | 613 | 15,3 | 582 | 15,5 | 31 | 11,7 | 0,039* |
|  | 25-34 years |  | 1786 | 44,5 | 1649 | 44,0 | 137 | 51,7 |  |
|  | 35-39 years |  | 1611 | 40,2 | 1514 | 40,4 | 97 | 36,6 |  |
|  | Mean(SD) in years |  | 31,6 | 5,9 | 31,6 | 5,9 | 31,6 | 5,4 |  |
| Time since diagnosis | <11 years |  | 1627 | 40,6 | 1508 | 40,3 | 119 | 44,9 | 0,289 |
|  | 11-15 years |  | 1385 | 34,5 | 1297 | 34,6 | 88 | 33,2 |  |
|  | >15 years |  | 998 | 24,9 | 940 | 25,1 | 58 | 21,9 |  |
|  | Mean(SD) in years |  | 12,4 | 4,5 | 12,4 | 4,5 | 11,9 | 4,5 |  |
| Age at questionnaire completion | 23-39 |  | 1089 | 27,2 | 1012 | 27,0 | 77 | 29,1 | 0,518 |
|  | 40-49 |  | 1915 | 47,8 | 1786 | 47,7 | 129 | 48,7 |  |
|  | 50-60 |  | 1006 | 25,1 | 947 | 25,3 | 59 | 22,3 |  |
|  | Mean(SD) in years |  | 44,5 | 7,5 | 44,5 | 7,5 | 43,9 | 7,1 |  |
| Sex | Male |  | 1549 | 38,6 | 1457 | 38,9 | 92 | 34,7 | 0,192 |
|  | Female |  | 2461 | 61,4 | 2288 | 61,1 | 173 | 65,3 |  |
| Partner status at time of questionnaire | No partner |  | 661 | 16,5 | 619 | 16,5 | 42 | 15,8 | 0,864 |
|  | Partner |  | 3333 | 83,1 | 3113 | 83,1 | 220 | 83,0 |  |
|  | Missing |  | 16 | 0,4 | 13 | 0,3 | 3 | 1,1 |  |
| Education (achieved) | No education or primary education | | 28 | 0,7 | 24 | 0,6 | 4 | 1,5 | 0,254 |
|  | Secondary education | | 266 | 6,6 | 251 | 6,7 | 15 | 5,7 |  |
|  | Secondary vocational education | | 1456 | 36,3 | 1357 | 36,2 | 99 | 37,4 |  |
|  | Higher (vocational) education | | 1374 | 34,3 | 1276 | 34,1 | 98 | 37,0 |  |
|  | University education | | 878 | 21,9 | 829 | 22,1 | 49 | 18,5 |  |
|  | Missing |  | 8 | 0,2 | 8 | 0,2 |  |  |  |
| Living status | Living together¹ |  | 3499 | 87,3 | 3269 | 87,3 | 230 | 86,8 | 0,923 |
|  | Living alone |  | 502 | 12,5 | 468 | 12,5 | 34 | 12,8 |  |
|  | Missing |  | 9 | 0,2 | 8 | 0,2 | 1 | 0,4 |  |
| Tumortype | Head and neck |  | 124 | 3,1 | 116 | 3,1 | 8 | 3,0 | n.a. |
|  | Digestive track, other | | 31 | 0,8 | 30 | 0,8 | 1 | 0,4 |  |
|  | Colon and rectal |  | 82 | 2,0 | 76 | 2,0 | 6 | 2,3 |  |
|  | Bone and soft tissue |  | 172 | 4,3 | 165 | 4,4 | 7 | 2,6 |  |
|  | Respiratory tract |  | 30 | 0,7 | 27 | 0,7 | 3 | 1,1 |  |
|  | Melanoma |  | 290 | 7,2 | 262 | 7,0 | 28 | 10,6 |  |
|  | Other |  | 11 | 0,3 | 10 | 0,3 | 1 | 0,4 |  |
|  | Germ cell |  | 692 | 17,3 | 653 | 17,4 | 39 | 14,7 |  |
|  | Breast |  | 944 | 23,5 | 887 | 23,7 | 57 | 21,5 |  |
|  | Female genitalia |  | 445 | 11,1 | 407 | 10,9 | 38 | 14,3 |  |
|  | Male genitalia |  | 6 | 0,1 | 5 | 0,1 | 1 | 0,4 |  |
|  | Urinary tract |  | 46 | 1,1 | 39 | 1,0 | 7 | 2,6 |  |
|  | Lymphoid hematological | | 591 | 14,7 | 556 | 14,8 | 35 | 13,2 |  |
|  | Myeloid hematological | | 148 | 3,7 | 140 | 3,7 | 8 | 3,0 |  |
|  | Thyroid gland |  | 248 | 6,2 | 230 | 6,1 | 18 | 6,8 |  |
|  | Central nervous system | | 150 | 3,7 | 142 | 3,8 | 8 | 3,0 |  |
| Primary treatment² | Chemotherapy | No | 1767 | 44,1 | 1634 | 43,6 | 133 | 50,2 | **0,041*** |
|  |  | Yes | 2239 | 55,8 | 2107 | 56,3 | 132 | 49,8 |  |
|  |  | Missing | 4 | 0,1 | 4 | 0,1 |  |  |  |
|  | Radiotherapy | No | 2104 | 52,5 | 1961 | 52,4 | 143 | 54,0 | 0,656 |
|  |  | Yes | 1902 | 47,4 | 1780 | 47,5 | 122 | 46,0 |  |
|  |  | Missing | 4 | 0,1 | 4 | 0,1 |  |  |  |
|  | Hormonal therapy | No | 3522 | 87,8 | 3285 | 87,7 | 237 | 89,4 | 0,440 |
|  |  | Yes | 484 | 12,1 | 456 | 12,2 | 28 | 10,6 |  |
|  |  | Missing | 4 | 0,1 | 4 | 0,1 |  |  |  |
|  | Targeted therapy | No | 3698 | 92,2 | 3451 | 92,1 | 247 | 93,2 | 0,635 |
|  |  | Yes | 308 | 7,7 | 290 | 7,7 | 18 | 6,8 |  |
|  |  | Missing | 4 | 0,1 | 4 | 0,1 |  |  |  |
|  | Surgery | No | 880 | 21,9 | 819 | 21,9 | 61 | 23,0 | 0,701 |
|  |  | Yes | 3126 | 78,0 | 2922 | 78,0 | 204 | 77,0 |  |
|  |  | Missing | 4 | 0,1 | 4 | 0,1 |  |  |  |
|  | Stem cell therapy | No | 3864 | 96,4 | 3606 | 96,3 | 258 | 97,4 | 0,494 |
|  |  | Yes | 142 | 3,5 | 135 | 3,6 | 7 | 2,6 |  |
|  |  | Missing | 4 | 0,1 | 4 | 0,1 |  |  |  |
| Tumor stage | I |  | 1726 | 43,0 | 1602 | 42,8 | 124 | 46,8 | 0,416 |
|  | II |  | 1063 | 26,5 | 992 | 26,5 | 71 | 26,8 |  |
|  | III |  | 573 | 14,3 | 536 | 14,3 | 37 | 14,0 |  |
|  | IV |  | 179 | 4,5 | 172 | 4,6 | 7 | 2,6 |  |
|  | Missing |  | 469 | 11,7 | 443 | 11,8 | 26 | 9,8 |  |
| Note: * p<0,05; ** p<0,01; na = not applicable; N= number; SD= standard deviation; ¹ Includes living with partner, parents, children, roommates or others; ² >100% as participants may have received multiple treatments. | | | | | | | | | |
