# Supplementary material for: Health-related quality of life of long-term adolescent and young adult (AYA) cancer survivors compared to a matched normative population: results of the SURVAYA study
Source: J Cancer Surviv. 2025 Jun 17;20(Suppl 1):628–51. doi: 10.1007/s11764-025-01818-0 (PMC13676661; doi:10.1007/s11764-025-01818-0)
Supplement: Supplementary file 4 [file 11764_2025_1818_MOESM4_ESM.docx]

**Appendix Table 2. Mean QoL scores per scale of AYA cancer survivors compared to matched normative population, per tumor type**

| Tumor types |  | **Global QoL** | | | | | | **Physical functioning** | | | | | | **Cognitive functioning** | | | | | | **Emotional functioning** | | | | | | **Role functioning** | | | | | | **Social functioning** | | | | | |
| --- | --- | --- | --- | --- | --- | --- | --- | --- | --- | --- | --- | --- | --- | --- | --- | --- | --- | --- | --- | --- | --- | --- | --- | --- | --- | --- | --- | --- | --- | --- | --- | --- | --- | --- | --- | --- | --- |
|  |  | AYA cancer survivors | | Normative population | | | p-value | AYA cancer survivors | | Normative population | | | p-value | AYA cancer survivors | | Normative population | | | p-value | AYA cancer survivors | | Normative population | | | p-value | AYA cancer survivors | | Normative population | | | p-value | AYA cancer survivors | | Normative population | | | p-value |
| Head and neck | N (observed) | 116 |  | | 665 |  |  | 116 |  | | 665 |  |  | 115 |  | | 665 |  |  | 115 |  | | 665 |  |  | 115 |  | | 665 |  |  | 115 |  | | 665 |  |  |
|  | Mean (SD) | 76,0 | 16,7 | | 75,7 | 17,9 |  | 92,4 | 13,3 | | 94,3 | 12,2 |  | 83,8 | 21,9 | | 92,2 | 15,7 |  | 82,2 | 20,4 | | 84,6 | 20,1 |  | 86,2 | 23,4 | | 91,5 | 19,6 |  | 89,1 | 21,7 | | 93,5 | 17,5 |  |
|  | Range (observed) | 25 | 100 | | 0 | 100 |  | 46,67 | 100 | | 0 | 100 |  | 16,67 | 100 | | 0 | 100 |  | 0 | 100 | | 0 | 100 |  | 0 | 100 | | 0 | 100 |  | 0 | 100 | | 0 | 100 |  |
|  | Mean difference | 0,3 |  | |  |  | 0,854 | -1,9 |  | |  |  | 0,126 | -8,4 |  | |  |  | <,001** | -2,3 |  | |  |  | 0,253 | -5,3 |  | |  |  | 0,024* | -4,4 |  | |  |  | 0,042* |
|  | Clinically relevant difference*** | Trivial |  | |  |  |  | Trivial |  | |  |  |  | Small |  | |  |  |  | Trivial |  | |  |  |  | Trivial |  | |  |  |  | Trivial |  | |  |  |  |
| Digestive tract, other | N (observed) | 30 |  | | 401 |  |  | 30 |  | | 401 |  |  | 30 |  | | 401 |  |  | 30 |  | | 401 |  |  | 30 |  | | 401 |  |  | 30 |  | | 401 |  |  |
|  | Mean (SD) | 75,6 | 19,8 | | 75,4 | 18,1 |  | 88,7 | 18,8 | | 95,5 | 10,1 |  | 83,3 | 21,0 | | 92,6 | 14,7 |  | 77,9 | 22,3 | | 85,3 | 19,3 |  | 83,3 | 28,4 | | 93,3 | 16,3 |  | 86,1 | 23,6 | | 94,3 | 15,7 |  |
|  | Range (observed) | 25 | 100 | | 0 | 100 |  | 20 | 100 | | 33,33 | 100 |  | 33,33 | 100 | | 0 | 100 |  | 33,33 | 100 | | 0 | 100 |  | 0 | 100 | | 0 | 100 |  | 16,67 | 100 | | 0 | 100 |  |
|  | Mean difference | 0,1 |  | |  |  | 0,972 | -6,8 |  | |  |  | 0,059 | -9,3 |  | |  |  | 0,023* | -7,4 |  | |  |  | 0,045* | -10,0 |  | |  |  | 0,067 | -8,2 |  | |  |  | 0,072 |
|  | Clinically relevant difference*** | Trivial |  | |  |  |  | Small |  | |  |  |  | Medium |  | |  |  |  | Small |  | |  |  |  | Small |  | |  |  |  | Small |  | |  |  |  |
| Colon and rectal | N (observed) | 76 |  | | 527 |  |  | 76 |  | | 527 |  |  | 75 |  | | 527 |  |  | 76 |  | | 527 |  |  | 76 |  | | 527 |  |  | 76 |  | | 527 |  |  |
|  | Mean (SD) | 72,1 | 18,2 | | 75,6 | 18,6 |  | 89,7 | 16,4 | | 94,0 | 12,3 |  | 80,0 | 26,0 | | 92,0 | 16,6 |  | 77,7 | 21,8 | | 84,7 | 20,6 |  | 81,1 | 27,0 | | 91,1 | 20,1 |  | 85,3 | 24,0 | | 93,2 | 17,2 |  |
|  | Range (observed) | 25 | 100 | | 0 | 100 |  | 6,67 | 100 | | 20 | 100 |  | 0 | 100 | | 0 | 100 |  | 0 | 100 | | 0 | 100 |  | 0 | 100 | | 0 | 100 |  | 0 | 100 | | 0 | 100 |  |
|  | Mean difference | -3,4 |  | |  |  | 0,134 | -4,3 |  | |  |  | 0,032* | -12,0 |  | |  |  | <,001** | -6,9 |  | |  |  | 0,007** | -9,9 |  | |  |  | 0,003** | -7,9 |  | |  |  | 0,007** |
|  | Clinically relevant difference*** | Trivial |  | |  |  |  | Trivial |  | |  |  |  | Medium |  | |  |  |  | Small |  | |  |  |  | Small |  | |  |  |  | Small |  | |  |  |  |
| Bone and soft tissue | N (observed) | 165 |  | | 650 |  |  | 165 |  | | 650 |  |  | 164 |  | | 650 |  |  | 165 |  | | 650 |  |  | 165 |  | | 650 |  |  | 165 |  | | 650 |  |  |
|  | Mean (SD) | 77,3 | 15,2 | | 75,8 | 18,6 |  | 87,2 | 18,1 | | 94,5 | 11,0 |  | 81,3 | 21,7 | | 92,1 | 15,1 |  | 80,4 | 19,9 | | 84,1 | 20,3 |  | 81,3 | 25,1 | | 91,7 | 19,1 |  | 87,6 | 22,2 | | 93,7 | 16,5 |  |
|  | Range (observed) | 16,67 | 100 | | 0 | 100 |  | 20 | 100 | | 33,33 | 100 |  | 0 | 100 | | 0 | 100 |  | 0 | 100 | | 0 | 100 |  | 0 | 100 | | 0 | 100 |  | 0 | 100 | | 0 | 100 |  |
|  | Mean difference | 1,5 |  | |  |  | 0,292 | -7,3 |  | |  |  | <,001** | -10,8 |  | |  |  | <,001** | -3,8 |  | |  |  | 0,033* | -10,4 |  | |  |  | <,001** | -6,1 |  | |  |  | 0,001** |
|  | Clinically relevant difference*** | Trivial |  | |  |  |  | Small |  | |  |  |  | Medium |  | |  |  |  | Trivial |  | |  |  |  | Small |  | |  |  |  | Small |  | |  |  |  |
| Respiratory tract | N (observed) | 27 |  | | 582 |  |  | 27 |  | | 582 |  |  | 27 |  | | 582 |  |  | 27 |  | | 582 |  |  | 27 |  | | 582 |  |  | 27 |  | | 582 |  |  |
|  | Mean (SD) | 70,7 | 19,5 | | 75,2 | 18,3 |  | 80,7 | 21,3 | | 94,1 | 11,9 |  | 82,7 | 18,8 | | 91,5 | 16,5 |  | 76,5 | 19,8 | | 83,0 | 21,2 |  | 72,2 | 33,7 | | 91,4 | 19,3 |  | 79,6 | 30,8 | | 93,8 | 16,7 |  |
|  | Range (observed) | 16,67 | 100 | | 0 | 100 |  | 20 | 100 | | 0 | 100 |  | 50 | 100 | | 0 | 100 |  | 25 | 100 | | 0 | 100 |  | 0 | 100 | | 0 | 100 |  | 0 | 100 | | 0 | 100 |  |
|  | Mean difference | -4,5 |  | |  |  | 0,211 | -13,4 |  | |  |  | 0,003** | -8,8 |  | |  |  | 0,007** | -6,5 |  | |  |  | 0,122 | -19,2 |  | |  |  | 0,007** | -14,1 |  | |  |  | 0,025* |
|  | Clinically relevant difference*** | Small |  | |  |  |  | Small |  | |  |  |  | Small |  | |  |  |  | Small |  | |  |  |  | Medium |  | |  |  |  | Medium |  | |  |  |  |
| Melanoma | N (observed) | 259 |  | | 502 |  |  | 262 |  | | 503 |  |  | 258 |  | | 502 |  |  | 258 |  | | 502 |  |  | 257 |  | | 503 |  |  | 258 |  | | 502 |  |  |
|  | Mean (SD) | 78,3 | 17,8 | | 76,3 | 18,2 |  | 94,5 | 12,8 | | 93,9 | 12,2 |  | 89,6 | 17,4 | | 91,5 | 16,6 |  | 84,1 | 19,2 | | 83,4 | 21,0 |  | 91,2 | 21,1 | | 91,9 | 18,8 |  | 93,8 | 14,9 | | 93,2 | 17,3 |  |
|  | Range (observed) | 8,33 | 100 | | 0 | 100 |  | 13,33 | 100 | | 26,67 | 100 |  | 0 | 100 | | 0 | 100 |  | 0 | 100 | | 0 | 100 |  | 0 | 100 | | 0 | 100 |  | 33,33 | 100 | | 0 | 100 |  |
|  | Mean difference | 2,0 |  | |  |  | 0,144 | 0,7 |  | |  |  | 0,481 | -1,9 |  | |  |  | 0,141 | 0,8 |  | |  |  | 0,620 | -0,7 |  | |  |  | 0,656 | 0,6 |  | |  |  | 0,651 |
|  | Clinically relevant difference*** | Trivial |  | |  |  |  | Trivial |  | |  |  |  | Trivial |  | |  |  |  | Trivial |  | |  |  |  | Trivial |  | |  |  |  | Trivial |  | |  |  |  |
| Germ cell | N (observed) | 652 |  | | 277 |  |  | 652 |  | | 277 |  |  | 653 |  | | 277 |  |  | 653 |  | | 277 |  |  | 653 |  | | 277 |  |  | 653 |  | | 277 |  |  |
|  | Mean (SD) | 77,2 | 16,9 | | 77,0 | 16,7 |  | 95,9 | 9,1 | | 97,3 | 7,0 |  | 83,4 | 21,6 | | 92,5 | 15,6 |  | 83,4 | 18,7 | | 86,2 | 19,6 |  | 89,9 | 20,6 | | 95,1 | 14,2 |  | 94,3 | 16,0 | | 95,6 | 13,9 |  |
|  | Range (observed) | 0 | 100 | | 0 | 100 |  | 33,33 | 100 | | 40 | 100 |  | 0 | 100 | | 0 | 100 |  | 0 | 100 | | 0 | 100 |  | 0 | 100 | | 16,67 | 100 |  | 0 | 100 | | 0 | 100 |  |
|  | Mean difference | 0,2 |  | |  |  | 0,871 | -1,4 |  | |  |  | 0,012* | -9,1 |  | |  |  | <,001** | -2,8 |  | |  |  | 0,039* | -5,1 |  | |  |  | <,001** | -1,3 |  | |  |  | 0,223 |
|  | Clinically relevant difference*** | Trivial |  | |  |  |  | Trivial |  | |  |  |  | Medium |  | |  |  |  | Trivial |  | |  |  |  | Trivial |  | |  |  |  | Trivial |  | |  |  |  |
| Breast | N (observed) | 879 |  | | 304 |  |  | 886 |  | | 304 |  |  | 885 |  | | 304 |  |  | 885 |  | | 304 |  |  | 883 |  | | 304 |  |  | 882 |  | | 304 |  |  |
|  | Mean (SD) | 75,2 | 17,7 | | 74,9 | 19,5 |  | 90,0 | 13,6 | | 91,6 | 14,2 |  | 72,4 | 25,3 | | 90,9 | 16,8 |  | 76,8 | 21,0 | | 81,8 | 20,9 |  | 80,7 | 26,1 | | 88,2 | 22,3 |  | 86,6 | 22,9 | | 91,3 | 18,6 |  |
|  | Range (observed) | 8,33 | 100 | | 0 | 100 |  | 0 | 100 | | 20 | 100 |  | 0 | 100 | | 0 | 100 |  | 0 | 100 | | 0 | 100 |  | 0 | 100 | | 0 | 100 |  | 0 | 100 | | 16,67 | 100 |  |
|  | Mean difference | 0,3 |  | |  |  | 0,814 | -1,6 |  | |  |  | 0,086 | -18,5 |  | |  |  | <,001** | -4,9 |  | |  |  | <,001** | -7,5 |  | |  |  | <,001** | -4,6 |  | |  |  | <,001** |
|  | Clinically relevant difference*** | Trivial |  | |  |  |  | Trivial |  | |  |  |  | Large |  | |  |  |  | Trivial |  | |  |  |  | Small |  | |  |  |  | Trivial |  | |  |  |  |
| Female genitalia | N (observed) | 406 |  | | 303 |  |  | 407 |  | | 303 |  |  | 407 |  | | 303 |  |  | 407 |  | | 303 |  |  | 407 |  | | 303 |  |  | 406 |  | | 303 |  |  |
|  | Mean (SD) | 75,3 | 17,8 | | 75,8 | 19,0 |  | 91,4 | 13,6 | | 91,6 | 15,1 |  | 78,3 | 23,6 | | 90,9 | 17,7 |  | 79,7 | 19,2 | | 82,0 | 20,9 |  | 83,1 | 26,2 | | 89,4 | 21,7 |  | 85,0 | 24,6 | | 92,5 | 17,6 |  |
|  | Range (observed) | 16,67 | 100 | | 16,67 | 100 |  | 26,67 | 100 | | 20 | 100 |  | 0 | 100 | | 0 | 100 |  | 0 | 100 | | 0 | 100 |  | 0 | 100 | | 0 | 100 |  | 0 | 100 | | 0 | 100 |  |
|  | Mean difference | -0,5 |  | |  |  | 0,721 | -0,2 |  | |  |  | 0,837 | -12,6 |  | |  |  | <,001** | -2,3 |  | |  |  | 0,128 | -6,3 |  | |  |  | <,001** | -7,4 |  | |  |  | <,001** |
|  | Clinically relevant difference*** | Trivial |  | |  |  |  | Trivial |  | |  |  |  | Medium |  | |  |  |  | Trivial |  | |  |  |  | Small |  | |  |  |  | Small |  | |  |  |  |
| Urinary tract | N (observed) | 38 |  | | 524 |  |  | 39 |  | | 524 |  |  | 38 |  | | 524 |  |  | 38 |  | | 524 |  |  | 38 |  | | 524 |  |  | 38 |  | | 524 |  |  |
|  | Mean (SD) | 71,1 | 18,7 | | 75,2 | 18,8 |  | 88,0 | 15,8 | | 93,9 | 11,7 |  | 78,5 | 27,6 | | 92,0 | 15,6 |  | 75,9 | 25,3 | | 83,9 | 20,3 |  | 78,5 | 30,0 | | 91,2 | 19,5 |  | 86,0 | 22,8 | | 93,4 | 16,3 |  |
|  | Range (observed) | 8,33 | 100 | | 0 | 100 |  | 53,33 | 100 | | 33,33 | 100 |  | 0 | 100 | | 0 | 100 |  | 0 | 100 | | 0 | 100 |  | 0 | 100 | | 0 | 100 |  | 0 | 100 | | 0 | 100 |  |
|  | Mean difference | -4,1 |  | |  |  | 0,194 | -5,9 |  | |  |  | 0,028* | -13,4 |  | |  |  | 0,005** | -8,1 |  | |  |  | 0,021* | -12,7 |  | |  |  | 0,014* | -7,4 |  | |  |  | 0,056 |
|  | Clinically relevant difference*** | Small |  | |  |  |  | Small |  | |  |  |  | Medium |  | |  |  |  | Small |  | |  |  |  | Small |  | |  |  |  | Small |  | |  |  |  |
| Lymphoid hematological | N (observed) | 553 |  | | 587 |  |  | 556 |  | | 587 |  |  | 554 |  | | 587 |  |  | 555 |  | | 587 |  |  | 555 |  | | 587 |  |  | 553 |  | | 587 |  |  |
|  | Mean (SD) | 74,1 | 17,5 | | 74,9 | 18,4 |  | 91,9 | 13,6 | | 94,4 | 12,0 |  | 75,2 | 26,4 | | 91,3 | 16,4 |  | 79,1 | 21,2 | | 83,1 | 21,7 |  | 82,5 | 25,3 | | 90,8 | 20,9 |  | 86,9 | 22,0 | | 93,0 | 17,1 |  |
|  | Range (observed) | 8,33 | 100 | | 0 | 100 |  | 20 | 100 | | 20 | 100 |  | 0 | 100 | | 0 | 100 |  | 0 | 100 | | 0 | 100 |  | 0 | 100 | | 0 | 100 |  | 0 | 100 | | 0 | 100 |  |
|  | Mean difference | -0,7 |  | |  |  | 0,484 | -2,4 |  | |  |  | 0,001** | -16,2 |  | |  |  | <,001** | -4,0 |  | |  |  | 0,002** | -8,3 |  | |  |  | <,001** | -6,0 |  | |  |  | <,001** |
|  | Clinically relevant difference*** | Trivial |  | |  |  |  | Trivial |  | |  |  |  | Large |  | |  |  |  | Trivial |  | |  |  |  | Small |  | |  |  |  | Small |  | |  |  |  |
| Myeloid hematological | N (observed) | 139 |  | | 591 |  |  | 140 |  | | 591 |  |  | 140 |  | | 591 |  |  | 140 |  | | 591 |  |  | 139 |  | | 591 |  |  | 139 |  | | 591 |  |  |
|  | Mean (SD) | 71,2 | 20,5 | | 74,7 | 19,1 |  | 86,7 | 18,2 | | 93,7 | 12,1 |  | 76,5 | 23,6 | | 91,8 | 15,5 |  | 80,1 | 19,8 | | 83,7 | 20,2 |  | 75,1 | 30,4 | | 90,1 | 20,8 |  | 83,0 | 23,9 | | 92,8 | 17,1 |  |
|  | Range (observed) | 0 | 100 | | 0 | 100 |  | 20 | 100 | | 26,67 | 100 |  | 0 | 100 | | 0 | 100 |  | 0 | 100 | | 0 | 100 |  | 0 | 100 | | 0 | 100 |  | 0 | 100 | | 0 | 100 |  |
|  | Mean difference | -3,5 |  | |  |  | 0,059 | -7,0 |  | |  |  | <,001** | -15,3 |  | |  |  | <,001** | -3,6 |  | |  |  | 0,056 | -15,1 |  | |  |  | <,001** | -9,8 |  | |  |  | <,001** |
|  | Clinically relevant difference*** | Trivial |  | |  |  |  | Small |  | |  |  |  | Large |  | |  |  |  | Trivial |  | |  |  |  | Small |  | |  |  |  | Small |  | |  |  |  |
| Thyroid gland | N (observed) | 229 |  | | 448 |  |  | 230 |  | | 448 |  |  | 229 |  | | 448 |  |  | 229 |  | | 448 |  |  | 229 |  | | 448 |  |  | 229 |  | | 448 |  |  |
|  | Mean (SD) | 72,7 | 17,1 | | 74,2 | 19,0 |  | 90,1 | 15,6 | | 93,0 | 12,7 |  | 76,0 | 23,3 | | 91,3 | 16,3 |  | 76,7 | 21,3 | | 82,9 | 20,7 |  | 80,5 | 25,2 | | 89,8 | 20,7 |  | 86,8 | 22,0 | | 92,4 | 17,2 |  |
|  | Range (observed) | 8,33 | 100 | | 0 | 100 |  | 0 | 100 | | 33,33 | 100 |  | 0 | 100 | | 0 | 100 |  | 0 | 100 | | 0 | 100 |  | 0 | 100 | | 0 | 100 |  | 0 | 100 | | 16,67 | 100 |  |
|  | Mean difference | -1,5 |  | |  |  | 0,300 | -2,8 |  | |  |  | 0,017* | -15,3 |  | |  |  | <,001**' | -6,2 |  | |  |  | <,001** | -9,3 |  | |  |  | <,001** | -5,7 |  | |  |  | <,001** |
|  | Clinically relevant difference*** | Trivial |  | |  |  |  | Trivial |  | |  |  |  | Large |  | |  |  |  | Small |  | |  |  |  | Small |  | |  |  |  | Small |  | |  |  |  |
| Central nervous system | N (observed) | 141 |  | | 605 |  |  | 142 |  | | 606 |  |  | 141 |  | | 605 |  |  | 141 |  | | 605 |  |  | 141 |  | | 606 |  |  | 140 |  | | 605 |  |  |
|  | Mean (SD) | 72,6 | 15,9 | | 74,7 | 18,8 |  | 88,1 | 17,0 | | 94,2 | 11,9 |  | 66,8 | 28,7 | | 91,4 | 16,3 |  | 73,4 | 24,4 | | 82,8 | 21,3 |  | 72,9 | 28,4 | | 91,0 | 20,3 |  | 77,9 | 29,1 | | 93,4 | 16,7 |  |
|  | Range (observed) | 25 | 100 | | 0 | 100 |  | 13,33 | 100 | | 33,33 | 100 |  | 0 | 100 | | 0 | 100 |  | 0 | 100 | | 0 | 100 |  | 0 | 100 | | 0 | 100 |  | 0 | 100 | | 0 | 100 |  |
|  | Mean difference | -2,0 |  | |  |  | 0,235 | -6,1 |  | |  |  | <,001** | -24,6 |  | |  |  | <,001** | -9,4 |  | |  |  | <,001** | -18,1 |  | |  |  | <,001** | -15,5 |  | |  |  | <,001** |
|  | Clinically relevant difference*** | Trivial |  | |  |  |  | Small |  | |  |  |  | Large |  | |  |  |  | Small |  | |  |  |  | Small |  | |  |  |  | Large |  | |  |  |  |
| Note: * p<0,05; ** p<0,01; *** Based on Cocks et al. 2011.; N= number; SD= standard deviation; orange = trivial; yellow = small; green = medium; blue = large. | | | | | | | | | | | | | | | | | | | | | | | | | | | | | | | | | | | | | |
